# Supplementary material for: Whole-Genome Analysis of Human Papillomavirus Type 16 Prevalent in Japanese Women with or without Cervical Lesions
Source: Viruses. 2019 Apr 16;11(4):350. doi: 10.3390/v11040350 (PMC6520816; doi:10.3390/v11040350)
Supplement: Supplementary file 1 [file viruses-11-00350-s001.zip › supplementary material/viruses_TableS1.docx]

**Table S1**

HPV16 whole-genome sequences determined in this study

____________________________________________________________________________________

ID Stage Age Length (bp) Variant Other HPV Accession no.

____________________________________________________________________________________

#001 NILM 27 7904 A5 51, 52 LC456180

#002 NILM 38 7905 A4 52, 58 LC456181

#003 NILM 32 7906 A4 - LC456182

#004 NILM 43 7905 A4 66 LC456198

#005 NILM 34 7905 A5 51 LC456183

#006 NILM 32 7906 A4 31, 54, 68 LC456184

#007 NILM 49 7905 A3 - LC456185

#008 NILM 30 7906 A4 58 LC456199

#009 NILM 44 7906 A5 - LC456186

#010 NILM 32 7905 A4 - LC456200

#011 NILM 27 7966* A1 - LC456187

#012 NILM 69 7904 A5 44 LC456188

#013 NILM 35 7905 A4 - LC456189

#014 NILM 38 7908 D3 - LC456190

#015 NILM 31 7906 A4 - LC456201

#016 NILM 24 7906 A4 18, 59 LC456191

#017 NILM 62 7973* A1 - LC456192

#018 NILM 34 7904 A5 - LC456193

#019 NILM 38 7905 A4 31 LC456194

#020 NILM 37 7904 A5 - LC456195

#021 NILM 33 7905 A5 - LC456196

#022 NILM 30 7906 A1 - LC456197

#023 CIN1 31 7905 A4 - LC368952

#024 CIN1 71 7908 D1 - LC368953

#025 CIN1 28 7906 A5 18, 31 LC368954

#026 CIN1 30 7905 A1 - LC368955

#027 CIN1 31 7905 A4 - LC368960

#028 CIN1 51 7906 A1 58 LC368956

#029 CIN1 68 7905 A4 - LC368957

#030 CIN1 23 7904 A5 39, 52 LC368958

#031 CIN1 31 7905 A2 - LC368961

#032 CIN1 38 7905 A2 - LC368962

#033 CIN1 35 7905 A4 - LC368963

#034 CIN1 46 7968* A1 - LC368964

#035 CIN1 42 7903 C 56 LC368959

#036 CIN2 40 7905 A4 - LC368969

#037 CIN2 36 7905 A4 - LC368970

#038 CIN2 43 7905 A1 - LC368971

#039 CIN2 50 7905 A4 - LC368972

#040 CIN2 38 7905 A1 - LC368973

#041 CIN2 30 7906 A1 - LC368968

#042 CIN2 30 7906 A1 - LC456637

#043 CIN2 41 7876** A4 82 LC368966

#044 CIN2 29 7905 A5 - LC368965

#045 CIN2 45 7906 A1 52,53,58,68 LC368967

#046 CIN3 33 7905 A5 - LC368974

#047 CIN3 34 7905 A1 - LC368983

#048 CIN3 31 7971* A1 - LC368984

#049 CIN3 33 7905 A5 - LC368985

#050 CIN3 26 7968* A1 - LC368976

#051 CIN3 55 7904 A5 - LC368987

#052 CIN3 32 7905 A1 - LC368988

#053 CIN3 37 7906 A1 - LC368980

#054 CIN3 35 7905 A4 - LC368981

#055 CIN3 35 7905 A4 - LC368975

#056 SCC 47 7905 A4 - LC368986

#057 CIN3 32 7906 A1 - LC368977

#058 CIN3 45 7905 A4 - LC368978

#059 CIN3 37 7907 A1 - LC368979

#060 CIN3 50 7884*** A4 - LC368982

#061 SCC 35 7905 A4 - LC456606

#062 SCC 33 7904 A4 - LC456607

#063 SCC 36 7906 A4 - LC456608

#064 SCC 46 7905 A1 - LC456609

#065 SCC 36 7906 A4 - LC456610

#066 SCC 29 7905 A4 - LC456611

#067 SCC 35 7863*** A4 - LC456612

#068 SCC 36 7905 A4 - LC456613

#069 SCC 47 7906 A4 - LC456614

#070 SCC 35 7906 A4 - LC456615

#071 SCC 37 7905 A4 - LC456616

#072 SCC 34 7906 A4 - LC456617

#073 SCC 29 7908 D3 - LC456618

#074 SCC 64 7905 A5 - LC456619

#075 SCC 29 7905 A4 - LC456620

#076 SCC 39 7905 A4 - LC456621

#077 SCC 37 7905 A4 - LC456622

#078 SCC 80 7909 A4 - LC456623

#079 SCC 41 7895** A4 - LC456624

#080 SCC 32 7905 A4 53 LC368989

#081 SCC 35 7905 A4 - LC368990

#082 SCC 38 7905 A3 - LC368991

#083 SCC 42 7905 A4 - LC368992

#084 SCC 34 7905 A4 82 LC368995

#085 SCC 48 7905 A4 - LC368996

#086 SCC 60 7905 A4 - LC368997

#087 SCC 32 7906 A1 - LC456628

#088 SCC 38 7906 A4 - LC456629

#089 SCC 29 7905 A4 - LC456630

#090 SCC 57 7905 A4 - LC456631

#091 SCC 53 7905 A3 - LC456632

#092 SCC 73 7905 A5 - LC456625

#093 SCC 60 7904 C 56 LC456626

#094 SCC 38 7906 A1 - LC456627

#095 Ad 44 7906 A4 - LC456633

#096 Ad 41 7905 A5 - LC456634

#097 Ad 48 7908 D2 - LC368993

#098 Ad 55 7905 A4 - LC368994

#099 PC 33 7905 A4 - LC456635

#100 NC 33 7905 A4 - LC456636

____________________________________________________________________________________

NILM, negative for intraepithelial lesion or malignancy; CIN, cervical intraepithelial neoplasia; SCC, squamous cell carcinoma; Ad, adenocarcinoma; PC, poorly differentiated carcinoma; NC, neuroendocrine carcinoma. *insertion in *E1*; **deletion in *E1*; ***deletion in *E2/E4*.

The following sequences are identical: #001, #012 and #030; #065, #072 and #095; #002 and #004; #066 and #071.
