# Supplementary material for: Whole-Genome Analysis of Human Papillomavirus Type 16 Prevalent in Japanese Women with or without Cervical Lesions
Source: Viruses. 2019 Apr 16;11(4):350. doi: 10.3390/v11040350 (PMC6520816; doi:10.3390/v11040350)
Supplement: Supplementary file 1 [file viruses_supple_Fig_mod.pdf › supplementary material/viruses_supple_Fig_mod.pdf]

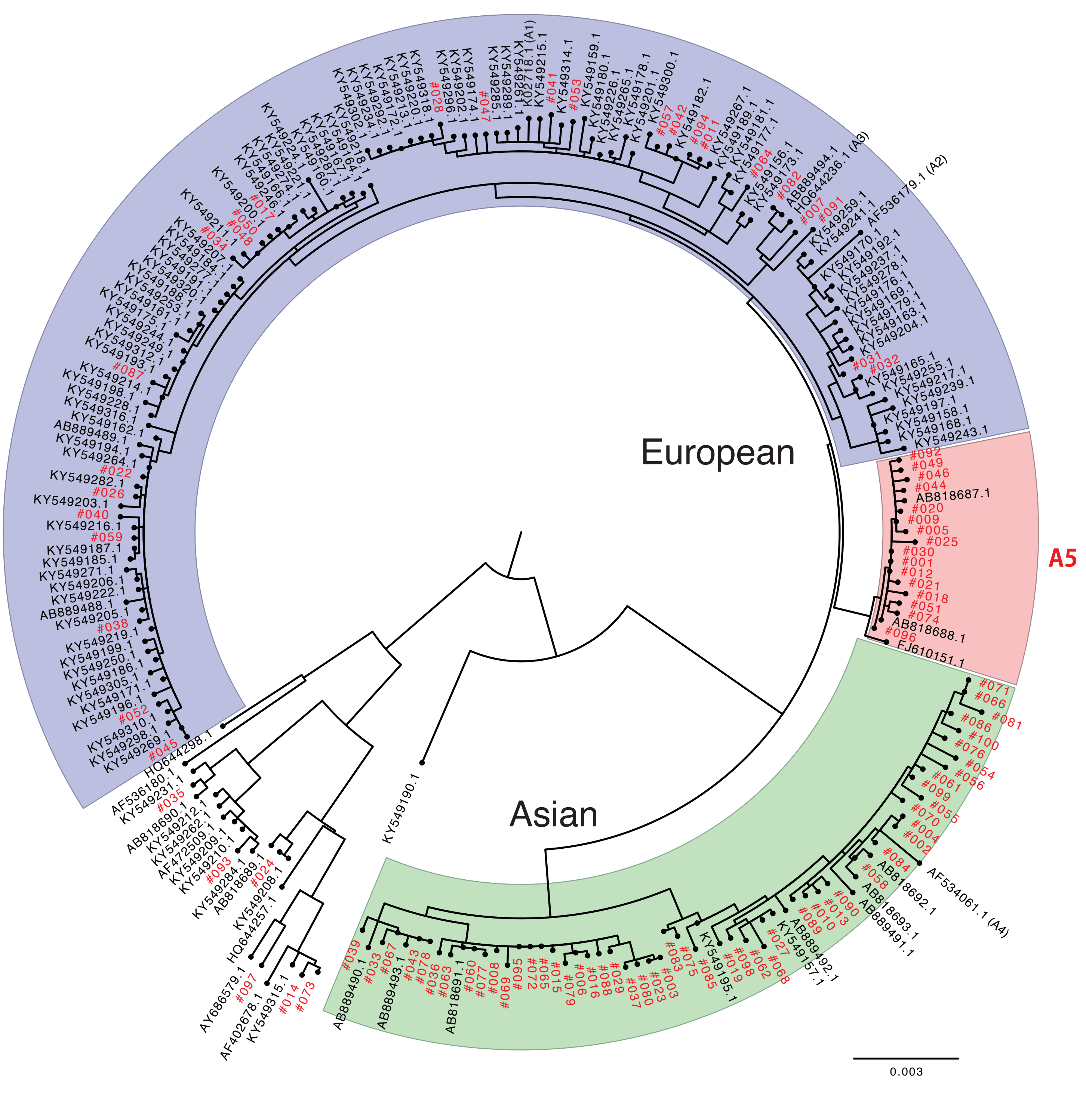

Figure S1. Maximum likelihood phylogenetic tree of HPV16 whole-genome sequences. The phylogenetic tree was constructed using whole-genome sequences of 114 Japanese isolates, 110 isolates from the Netherlands [37], one Thai isolate [33], and 10 references strains for HPV16 variant (sub) lineages. Japanese isolates determined in this study are indicated in red. Scale bar, nucleotide substitutions per site.
